# Quais os Valores de Normalidade mais Adequados para Monitorização Residencial da Pressão Arterial?

**DOI:** 10.36660/abc.20201109

**Published:** 2021-03-03

**Authors:** Audes D. M. Feitosa, Marco A. Mota-Gomes, Fernando Nobre, Decio Mion, Annelise M.G. Paiva, Fábio Argenta, Weimar K.S. Barroso, Roberto D. Miranda, Eduardo C. D. Barbosa, Andréa A. Brandão, Thiago S.V. Jardim, Paulo C.B.V. Jardim, Wilson Nadruz

**Affiliations:** 1 Universidade de Pernambuco Pronto Socorro Cardiológico de Pernambuco (PROCAPE) RecifePE Brasil Pronto Socorro Cardiológico de Pernambuco (PROCAPE), Universidade de Pernambuco, Recife, PE - Brasil; 2 Universidade Católica de Pernambuco Instituto UNICAP de Pesquisa Clínica RecifePE Brasil Instituto UNICAP de Pesquisa Clínica, Universidade Católica de Pernambuco, Recife, PE - Brasil; 3 Centro Universitário CESMAC MaceióAL Brasil Centro Universitário CESMAC, Maceió, AL - Brasil; 4 Universidade de São Paulo Ribeirão PretoSP Brasil Universidade de São Paulo, Ribeirão Preto, SP - Brasil; 5 Universidade de São Paulo Faculdade de Medicina Hospital das Clínicas São PauloSP Brasil Hospital das Clínicas, Faculdade de Medicina, Universidade de São Paulo, São Paulo, SP - Brasil; 6 Hospital Santa Rosa Programa de Residência Médica em Cardiologia CuiabáMT Brasil Hospital Santa Rosa, Programa de Residência Médica em Cardiologia, Cuiabá, MT - Brasil; 7 Universidade Federal de Goiás Faculdade de Medicina GoiâniaGO Brasil Liga de Hipertensão Arterial, Faculdade de Medicina, Universidade Federal de Goiás, Goiânia, GO - Brasil; 8 Universidade Federal de São Paulo Seção Cardiovascular São PauloSP Brasil Seção Cardiovascular, Disciplina de Geriatria, Universidade Federal de São Paulo, São Paulo, SP - Brasil; 9 Cardiometabolismo Hospital São Francisco e Santa Casa Misericórdia de Porto Alegre Serviço de Hipertensão Arterial Porto AlegreRS Brasil Liga de combate à Hipertensão Arterial de Porto Alegre, Serviço de Hipertensão Arterial e Cardiometabolismo Hospital São Francisco e Santa Casa Misericórdia de Porto Alegre, Porto Alegre, RS - Brasil; 10 Universidade do Estado do Rio de Janeiro Faculdade de Ciências Médicas Rio de JaneiroRJ Brasil Faculdade de Ciências Médicas, Universidade do Estado do Rio de Janeiro, Rio de Janeiro, RJ - Brasil; 11 Hospital do Coração de Goiás Ltda GoiâniaGO Brasil Hospital do Coração de Goiás Ltda, Goiânia, GO - Brasil; 12 Universidade Estadual de Campinas Faculdade de Ciências Médicas Departamento de Clínica Médica CampinasSP Brasil Departamento de Clínica Médica, Faculdade de Ciências Médicas, Universidade Estadual de Campinas, Campinas, SP - Brasil

**Keywords:** Hipertensão, Pressão Arterial, Valores Normais, Padrões de Referência, Automonitorização Pressão Arterial

## Introdução

O diagnóstico de hipertensão arterial (HA) habitualmente se baseia em medidas de pressão arterial (PA) obtidas no consultório. Esta estratégia, entretanto, pode subestimar ou superestimar a verdadeira prevalência de HA devido à presença de fenótipos alternativos, como HA mascarada e HA do avental branco. Neste contexto, as diretrizes atuais de HA têm recomendado, quando possível, a realização de medidas de PA fora do consultório, seja por meio da monitorização ambulatorial da PA (MAPA) ou da monitorização residencial da PA (MRPA), para a confirmação da HA e manuseio mais adequado do paciente.^1^^–^^4^

Valores de MRPA utilizados para definir HA começaram a ser propostos de forma mais consensual a partir do final da década de 1990. Em 1998, resultados de uma metanálise de 17 estudos envolvendo 5.422 participantes não tratados com medicações anti-hipertensivas sugeriram que valores elevados de MRPA fossem ≥ 137/89 mmHg ou ≥ 135/86 mmHg quando calculados a partir das médias + 2 desvios padrões ou dos percentis 95, respectivamente.^5^ No ano seguinte, a análise de 2.401 indivíduos normotensos no consultório estimou que valores elevados de MRPA fossem ≥ 137/85 mmHg a partir do cálculo dos percentis 95.^6^ Além disto, uma avaliação de 1.913 participantes (69% não tratados) do Estudo de Ohasama mostrou que valores de MRPA ≥ 137/84 mmHg se associaram com maior risco de morte após um seguimento médio de 5 anos.^7^ Os valores de referência propostos por estes estudos foram arredondados e embasaram a recomendação em diretrizes subsequentes de que medidas de MRPA fossem consideradas anormais quando maiores ou iguais a 135/85 mmHg.^8^^,^^9^ Estes valores de referência (135/85 mmHg) foram então incorporados à prática clínica e têm sido recomendados para determinar a presença de valores anormais de MRPA por diretrizes recentes de diversas sociedades^3^^,^^10^ incluindo a 7^a^ Diretriz Brasileira de HA, publicada em 2016,^1^ e as 6ᵃ Diretrizes Brasileiras de MAPA e 4ᵃ Diretrizes de MRPA, publicadas em 2018.^4^

Vários estudos publicados na última década avaliando indivíduos sem uso de medicações anti-hipertensivas têm apontado para a necessidade de revisão dos valores de referência de MRPA para diagnosticar HA^11^^–^^14^ Em 2012, Collde-Tuero e colaboradores relataram que valores de MRPA < 130/80 mmHg se associaram com menor desenvolvimento de lesões de órgão-alvo do que valores de MRPA < 135/85 mmHg em uma amostra de 466 indivíduos.^11^ Em 2017, um estudo coreano incluindo 256 participantes mostrou que valores de MRPA ≥ 130/80 mmHg apresentaram maior acurácia do que valores de MRPA ≥ 135/85 mmHg para detectar HA diagnosticada pela MAPA.^12^ Mais recentemente, em 2020, resultados de análise de regressão envolvendo 9.868 indivíduos brasileiros não tratados mostraram que valores de PA no consultório de 140/90 mmHg corresponderam a valores de 130/82 mmHg na MRPA.^13^ No que se refere a desfechos cardiovasculares, Niiranen et al.^14^ publicaram, em 2013, uma metanálise incluindo dados de 5.018 participantes não tratados de 5 países, a qual mostrou que valores de MRPA de 131,9/82,4 mmHg eram equivalentes a valores de PA no consultório de 140/90 mmHg na predição de eventos cardiovasculares.^14^ De maneira geral, estes estudos publicados na última década demonstram que valores de normalidade para MRPA são mais próximos de 130/80 mmHg do que 135/85 mmHg, dando suporte à mudança nos valores de referência de MRPA para 130/80 mmHg.

Diversas evidências obtidas em indivíduos sob tratamento anti-hipertensivo também indicam que valores de referência de MRPA mais baixos do que 135/85 mmHg sejam mais adequados para definir a presença de comportamento anormal da PA.^13^^,^^15^^–^^17^ Resultados de análise de regressão envolvendo 10.069 brasileiros tratados mostraram que valores de MRPA de 131/82 mmHg são equivalentes a valores da PA no consultório de 140/90 mmHg.^13^ No Estudo de Ohasama, a avaliação de 700 pacientes tratados mostrou que a incidência de acidente vascular encefálico foi maior em indivíduos com valores de MRPA entre 125/80 e 134/84 mmHg do que nos indivíduos com valores de MRPA < 115/75 mmHg após um seguimento médio de 11,9 anos, indicando que pacientes com valores de MRPA mais baixos que 135/85 mmHg ainda podem apresentar risco aumentado para desfechos vasculares desfavoráveis.^15^ Em outro estudo avaliando 3,5^18^ pacientes japoneses sob tratamento anti-hipertensivo, Asayama et al.,^16^ mostraram que os indivíduos que atingiram valores de MRPA sistólica menores que 131,6 mmHg apresentaram menor risco de desfechos cardiovasculares desfavoráveis.^16^ Recentemente, Coll-de-Tuero et al.^17^ mostraram que dentre os pacientes com PA elevada no consultório sem lesão de órgão-alvo, aqueles com valores de MRPA < 130/80 mmHg tiveram mortalidade similar a indivíduos normotensos no consultório, enquanto aqueles com valores de MRPA < 135/85 mmHg tiveram maior mortalidade.^17^ Em conjunto, as evidências obtidas em pacientes sob tratamento anti-hipertensivo dão suporte adicional à recomendação de que valores ≥ 130/80 mmHg sejam utilizados para detectar indivíduos com comportamento anormal da MRPA.

Por fim, consideramos válido ressaltar que a MRPA e a MAPA na vigília não devem ser consideradas medidas de PA iguais. A MAPA na vigília mede a PA durante as atividades habituais do paciente, incluindo trabalho, transporte, refeições, e em momentos de repouso e estresse. Por outro lado, a MRPA é derivada de um protocolo rígido em que o paciente faz as medidas apenas em ambiente calmo, após repouso de pelo menos 3 minutos, ao amanhecer e ao anoitecer, antes das refeições (ou 2 horas após o jantar), de bexiga vazia e antes do uso das medicações anti-hipertensivas (se for o caso). Neste contexto, é comum que os valores de MAPA na vigília sejam ligeiramente mais altos do que de MRPA.^18^ Portanto, pode-se afirmar que a MAPA na vigília e a MRPA são medidas distintas da PA, e consequentemente, podem apresentar valores de normalidade diferentes.

## Conclusão

Com base nas evidências acima, a Diretriz Brasileira de HA 2020^19^ recomenda que os valores de anormalidade de MRPA passem a ser ≥ 130/80 mmHg, em substituição aos valores ≥135/85 mmHg recomendados previamente pela 7^a^ Diretriz Brasileira de HA^1^ e pelas 6ᵃ Diretrizes de Monitorização Ambulatorial da Pressão Arterial e 4ᵃ Diretrizes de Monitorização Residencial da Pressão Arterial.^4^
