# Supplementary material for: Quais os Valores de Normalidade mais Adequados para Monitorização Residencial da Pressão Arterial?
Source: Arq Bras Cardiol. 2021 Mar 3;116(3):501–3. [Article in Portuguese] doi: 10.36660/abc.20201109 (PMC8159570; doi:10.36660/abc.20201109)
Supplement: Translation

# Translation: What are the Optimal Reference Values for Home Blood Pressure Monitoring?

**Authors:** Audes D. M. Feitosa, Marco A. Mota-Gomes, Fernando Nobre, Decio Mion, Annelise M.G. Paiva, Fábio Argenta, Weimar K.S. Barroso, Roberto D. Miranda, Eduardo C. D. Barbosa, Andréa A. Brandão, Thiago S.V. Jardim, Paulo C.B.V. Jardim, Wilson Nadruz

**Affiliations:** 1 RecifePE Brazil Pronto Socorro Cardiológico de Pernambuco (PROCAPE), Universidade de Pernambuco, Recife, PE - Brazil; 2 RecifePE Brazil Instituto UNICAP de Pesquisa Clínica, Universidade Católica de Pernambuco, Recife, PE - Brazil; 3 MaceióAL Brazil Centro Universitário CESMAC, Maceió, AL - Brazil; 4 Ribeirão PretoSP Brazil Universidade de São Paulo, Ribeirão Preto, SP - Brazil; 5 São PauloSP Brazil Hospital das Clínicas, Faculdade de Medicina, Universidade de São Paulo, São Paulo, SP - Brazil; 6 CuiabáMT Brazil Hospital Santa Rosa, Programa de Residência Médica em Cardiologia, Cuiabá, MT - Brazil; 7 GoiâniaGO Brazil Liga de Hipertensão Arterial, Faculdade de Medicina, Universidade Federal de Goiás, Goiânia, GO - Brazil; 8 São PauloSP Brazil Seção Cardiovascular, Disciplina de Geriatria, Universidade Federal de São Paulo, São Paulo, SP - Brazil; 9 Porto AlegreRS Brazil Liga de combate à Hipertensão Arterial de Porto Alegre, Serviço de Hipertensão Arterial e Cardiometabolismo Hospital São Francisco e Santa Casa Misericórdia de Porto Alegre, Porto Alegre, RS - Brazil; 10 Rio de JaneiroRJ Brazil Faculdade de Ciências Médicas, Universidade do Estado do Rio de Janeiro, Rio de Janeiro, RJ - Brazil; 11 GoiâniaGO Brazil Hospital do Coração de Goiás Ltda, Goiânia, GO - Brazil; 12 CampinasSP Brazil Departamento de Clínica Médica, Faculdade de Ciências Médicas, Universidade Estadual de Campinas, Campinas, SP - Brazil

**Keywords:** Hypertension, Blood Pressure, Reference Values, Reference Standards, Blood Pressure Self-Monitoring

## Introduction

The diagnosis of hypertension usually relies on office blood pressure (BP) measures. However, this approach might underestimate or overestimate the true prevalence of hypertension due to the presence of alternative BP phenotypes, such as masked hypertension and white-coat hypertension. In this regard, current hypertension guidelines have recommended the evaluation of out-of-office BP by ambulatory BP monitoring (ABPM) or home blood pressure monitoring (HBPM), when these techniques are available, to confirm the diagnosis and to provide a more adequate management of hypertension.^1^^–^^4^

HBPM reference values used to define hypertension have been more consistently suggested since the end of 1990s. In 1998, results of a meta-analysis including data from 17 studies and 5,422 untreated participants suggested that elevated HBPM values should be ≥ 137/89 mmHg or ≥ 135/86 mmHg based on analysis of means + 2 standard deviations or ninety-fifth percentiles, respectively.^5^ In 1999, the analysis of the ninety-fifth percentiles of HBPM values from 2,401 normotensive individuals at the office estimated that elevated HBPM values were ≥ 137/85 mmHg.^6^ Furthermore, the evaluation of 1,913 individuals (69% not treated with antihypertensive medications) from the Ohasama Study showed that HBPM values ≥ 137/84 mmHg were associated with greater risk of death after 5 years of follow-up.^7^ Subsequent hypertension guidelines rounded the reference values suggested by these latter studies and recommended that abnormal HBPM measures should be considered when ≥ 135/85 mmHg.^8^^,^^9^ HBPM thresholds of 135/85 mmHg were then incorporated into clinical practice, and they have been used to define abnormal HBPM values by hypertension guidelines from various societies,^3^^,^^10^ including the Seventh Brazilian Hypertension Guidelines published in 2016^1^ and the Sixth Brazilian Guidelines of ABPM and Fourth Brazilian Guidelines of HBPM published in 2018.^4^

Several reports published in the last decade evaluating individuals not using antihypertensive medications have suggested that HBPM reference values used to define hypertension should be reviewed.^11^^–^^14^ In 2012, Coll-de-Tuero et al. reported that HBPM values < 130/80 mmHg were associated with lower risk of development of end-organ damage than HBPM values < 135/85 mmHg in a sample of 466 individuals.^11^ In 2017, a Korean study evaluating 256 participants found that HBPM values ≥ 130/80 mmHg had greater accuracy than HBPM values ≥ 135/85 mmHg to detect hypertension, considering ABPM measurements as a reference.^12^ More recently, in 2020, results of regression analysis including 9,868 untreated Brazilian participants showed that office BP values of 140/90 mmHg corresponded to HBPM values of 130/82 mmHg.^13^ Regarding long-term outcomes, Niiranen et al.^14^ published, in 2013, a meta-analysis including data from 5,018 untreated individuals from 5 countries, which showed that HBPM values of 131.9/82.4 mmHg were equivalent to office BP values of 140/90 mmHg in predicting cardiovascular events.^14^ Overall, these studies demonstrated that normal HBPM values are actually closer to 130/80 mmHg than 135/85 mmHg, thus providing support for changing HBPM reference values from 135/85 mmHg to 130/80 mmHg.

Numerous reports evaluating individuals using antihypertensive medications have also indicated that HBPM values lower than 135/85 mmHg are more adequate to define the presence of high BP levels.^13^^,^^15^^–^^17^ Results of regression analysis including data from 10,069 treated Brazilian participants showed that HBPM values of 131/82 mmHg were equivalent to office BP values of 140/90 mmHg.^13^ The evaluation of 700 treated hypertensive patients from the Ohasama Study demonstrated that the incidence of stroke was greater in patients with HBPM values ranging between 125/80 and 134/84 mmHg than in those with HBPM values < 115/75 mmHg after a mean follow-up of 11.9 years, indicating that patients with HBPM values lower than 135/85 mmHg might still have greater risk of adverse cardiovascular events.^15^ In another study evaluating 3,518 treated Japanese patients, Asayama et al.^16^ found that individuals who achieved systolic HBPM values lower than 131.6 mmHg had lower risk of presenting adverse cardiovascular outcomes.^16^ Recently, Coll-de-Tuero et al.^17^ reported that, among treated patients with high office BP levels but no sign of end-organ damage, those with HBPM values < 130/80 mmHg had mortality similar to individuals with normal office BP levels, while individuals with HBPM values <135/85 mmHg had greater mortality.^17^ In general, these data obtained in treated hypertensive patients provide additional support to the idea that HBPM ≥ 130/80 mmHg should be used do define individuals with elevated BP levels.

Finally, we believe it is worth mentioning that HBPM and daytime ABPM should not be considered as equivalent measures. Daytime ABPM measures BP while the studied individuals are performing their regular activities at work, in transportation, or during meals and when they are at rest or under stress. Conversely, HBPM values are derived from a strict protocol where the studied individuals measure their BP in a quiet environment, after at least 3 minutes of rest, in the morning and in the evening, before using antihypertensive medications or having meals (or 2 hours after dinner), and with empty bladder. In this context, it is common that daytime ABPM values are slightly greater than HBPM values.^18^ Therefore, it can be stated that daytime ABPM and HBPM are distinct measures, and they may have different reference values.

## Conclusion

Based on the aforementioned evidence, the Brazilian Guidelines of Hypertension 2020^19^ recommend that HBPM values should be considered abnormal when they are greater or equal to 130/80 mmHg, thus substituting the previous thresholds (≥ 135/85 mmHg) recommended by the Seventh Brazilian Hypertension Guidelines,^1^ the Sixth Brazilian Guidelines of ABPM, and the Fourth Brazilian Guidelines of HBPM.^4^
